# USP2 Inhibits Lung Cancer Pathogenesis by Reducing ARID2 Protein Degradation via Ubiquitination

**DOI:** 10.1155/2022/1525216

**Published:** 2022-12-15

**Authors:** Lihuan Zhu, Zhizhong Chen, Tianxing Guo, Wenshu Chen, Lilan Zhao, Lingwen Guo, Xiaojie Pan

**Affiliations:** ^1^Department of Thoracic Surgery, Shengli Clinical Medical College of Fujian Medical University, Fujian Provincial Hospital, Fuzhou 350001, China; ^2^Department of Pathology, Shengli Clinical Medical College of Fujian Medical University, Fujian Provincial Hospital, Fuzhou 350001, China

## Abstract

**Background:**

Ubiquitination is an important regulator in physiological and pathological conditions. Ubiquitin-specific protease 2 (USP2), as a member of the USP family, exhibits oncogenic effects in multiple malignancies. However, the exact role of USP2 has not been well clarified in lung cancer pathogenesis and progression. Therefore, we aimed to further investigate the regulatory roles of USP2 in lung cancer in this study.

**Methods:**

Firstly, immunoprecipitation-Mass Spectrometry (IP-MS), Co-immunoprecipitation (Co-IP), combined with immunofluorescent colocalization method, was conducted for USP2 protein interaction analysis in lung cancer cell lines. qRT-PCR, Western blot, and immunohistochemistry assays explored the USP2 expression pattern and USP2/ARID2- (AT-rich interactive domain 2-) specific shRNAs and overexpression vectors. Co-IP assays were designed to validate USP2-ARID2 protein interaction. Further functional studies including CHX chase assay, transwell assay, and wound healing assay were subsequently applied to evaluate the impact of USP2 modulation on lung cancer cells.

**Results:**

USP2 suppression was characteristic in lung cancer cell line models and lung cancer samples. USP2 and ARID2 demonstrated protein-protein interaction and overlapping localization in cancer cell models. Functional experiments suggested USP2 inhibited lung cancer cell invasion and migration by reducing ARID2 protein degradation. Subsequent ubiquitination assays indicated ARID2 protein degradation via the ubiquitination was significantly reduced by USP2 interaction.

**Conclusions:**

Our study provided novel insight that USP2 might suppress lung cancer by reducing ARID2 protein degradation via ubiquitination.

## 1. Introduction

Ubiquitination is one of the most crucial cellular posttranscriptional regulatory pathways in human physiology and pathology. Ubiquitination includes the binding of ubiquitin (Ub) or ubiquitin-like proteins (UBLs) with a series of target proteins for subsequent degradation or signal transduction [1]. Previous investigators have demonstrated that ubiquitination plays important parts in a variety of processes in endocytosis, cell cycle regulation, and signal pathway activation and inactivation [2]. Other researchers have discovered nonproteolytic functions of ubiquitination including protein assembly, DNA repair, inflammation, and autophagy [3–5]. In the meantime, ubiquitination has also been linked to oncogenic pathway modulation in cancer pathogenesis. Studies have indicated the aberrant ubiquitination pathway is associated with the activation of the oncogenic pathway or tumor suppressor protein degradation, such as NF-*κΒ* and p53 [6, 7].

Ubiquitin-specific protease 2 (USP2), as a member of the USP family, belongs to the deubiquitinating enzyme (DUB) superfamily. It plays an important role in cellular ubiquitination. USP2 has been detected in various human tissues [8–10], and it participates in multiple physiological and pathological processes via the deubiquitination of multiple regulator proteins [11–13]. Notably, USP2 has also been intensively studied in cancer pathogenesis. Previous studies have demonstrated aberrant expression of USP2 in multiple malignancies including glioma, prostate cancer, and bladder carcinoma [14–16]. USP2 has been shown to enhance cancer cell proliferative and invasive capabilities by stabilizing and elevating murine double minute 2 (MDM2) protein, which subsequently promotes p53 protein elimination [17, 18]. Other studies also indicated that USP2 augments the cancer cell cycle by interacting with cyclin D1 and reducing its protein degradation by ubiquitination [19].

AT-rich interactive domain 2 (ARID2) is a member of AT-rich interactive domain (ARID)-containing family, and ARID2 could affect chromatin structure modification, transcriptional regulation, and cell cycle. The regulatory function of ARID2 in tumors has been widely reported. ARID2 was considered a tumor suppressor in early-onset sporadic rectal cancer through inhibiting tumor growth in vivo, cell migration, and viability [20]. Meanwhile, ARID2 suppression promoted oral cancer progression by upregulating cytokeratin 8 and 18 and *β*-4 integrin expression [21]. In addition, knockdown of ARID2 accelerated tumor progression in lung cancer and ARID2 deficiency was associated with higher sensitivity to chemotherapy [22]. However, if USP could regulate lung cancer through targeting ARID2 has not been reported.

Despite current advances in USP2-related cancer research, the exact significance of the USP2-associated regulatory network of signal pathways in lung cancer cells is limited. Therefore, in this study, we evaluated the impact of USP2 and its molecular regulatory mechanisms in lung cancer pathogenesis and progression with lung cancer cell models and clinical samples. The study is aimed at providing novel insight into the roles of USP2 in future lung cancer research.

## 2. Methods

### 2.1. Cell Lines and Clinical Sample Collection

Lung cancer cell lines (NCI-H1975, NCI-H292, H1299, NCI-H1395, and A549) and normal human bronchial epithelial (HBE) cell line were acquired from American Type Culture Collection (ATCC). Dulbecco's modified Eagle's medium combined with 10% fetal bovine serum and 1% Pen/Strep was used for cell culture at 37°C under 5% CO_2_. Lung cancer biopsy clinical samples were retrospectively collected from the clinical center, and matched surrounding normal tissue (NT) samples were also harvested. This study was conducted following the Declaration of Helsinki. Informed consents were acquired from all enrolled patients in this study.

### 2.2. Antibodies

Primary antibodies for immunoblotting include anti-USP2 (#HPA006777) and anti-ARID2 (#SAB2702508) were acquired from Sigma-Aldrich (US); anti-beta-actin from Abcam (#ab8226); anti-Myc (#sc-40), anti-Flag (#sc-7945), and anti-His/anti-HA (#sc-8036) from Santa Cruz Biotechnology; and anti-ubiquitin and anti-GAPDH from Thermo Fisher.

### 2.3. qRT-PCR

Total RNA extraction was performed using TRIzol (#15596026, Invitrogen, Carlsbad, USA). Reverse transcriptase (#639538, TAKARA) along with oligo-dT primers was applied for reverse transcription. SYBR green qPCR Mix kit (#4309155, Thermo Fisher) was used for the qRT-PCR process. Primers used in this study are listed as follows. USP (forward: 5′-GTCTGGCTGGTCTTCGAAAC-3′, reverse: 5′-CTTCCATGAGGGCCGTGT-3′), ARID2 (forward: 5′-CCTGATGCACTAGCTGCGGTAA-3′, reverse: 5′-GGAGCAACATGCTGCGCTACAA-3′), E-Cadherin (forward: 5′-GCCTCCTGAAAAGAGAGTGGAAG-3′, reverse: 5′-TGGCAGTGTCTCTCCAAATCCG-3′), N-Cadherin (forward: 5′-CCTCCAGAGTTTACTGCCATGAC-3′, reverse: 5′-GTAGGATCTCCGCCACTGATTC-3′), Vimentin (forward: 5′-AGGCAAAGCAGGAGTCCACTGA-3′, reverse: 5′-ATCTGGCGTTCCAGGGACTCAT-3′), GAPDH (forward: 5′-GTCCATGCCATCACTGCCAC-3′, reverse: 5′-AAGGCTGTGGGCAAGGTCAT-3′).

### 2.4. Western Blot

Proteins separation was conducted using SDS-PAGE on 5% Tris-acetate gels and 4% Bis-Tris gels (#NW0412C, Thermo Fisher). Proteins were subsequently moved to polyvinylidene difluoride membranes. Samples were treated with 1 : 1000 primary antibodies overnight and followed with horseradish peroxidase-conjugated secondary antibody treatment (#7074, Cell Signaling) for 2 hours at room temperature. The membranes were imaged by a myECL imager (#62239, Thermo Fisher).

### 2.5. Immunofluorescent Colocalization Assay

Cells were placed in a confocal dish, fixed with 4% paraformaldehyde, treated with 1 x PBS with 0.1% Triton X-100 for 10 min, and then blocked using 1% goat serum at room temperature for 20 min. After incubating with the appropriate primary antibody at 4°C overnight and the secondary antibody for 1 h, images were captured by a Leica confocal microscope. The image data were processed and quantified to colocalization ratio by ImageJ software.

### 2.6. Lentiviral Vector Transfection

pLVX expression vector was purchased from Clontech (USA). Polymerase chain reaction (PCR) was applied using DNA template sequences. PCR products were purified using 1% agarose gel and double digested by BamHI and EcoRI along with empty pLVX vectors.

A ligation reaction was performed overnight between the vector and the purified PCR products by T4DNA ligase. A ligation product was further used for the E. coli DH5*α* competent cell transformation. Cell clones were subsequently placed into the ampicillin-containing-LB plate and were incubated overnight at 37°C. Positive clones were gathered, and the plasmid was extracted and sequenced (Shanghai Invitrogen Biotech Co., Ltd). Afterward, the lentiviral vectors were packaged and subsequently added into tumor cells with a multiplicity of infection (MOI) of 20. The vector's titer was set as 1 × 10^9^/ml. The fluorescent protein expression level was quantified, and transfection efficiency was evaluated 24-48 h post-transfection.

### 2.7. Co-Immunoprecipitation (Co-IP)

Whole-cell lysates (WCL 400 *μ*g) were treated overnight with 3 *μ*g of anti-Flag/anti-Myc/anti-His/anti-HA antibody or IgG control antibody in TBS buffer (40 mM Tris-HCl pH 7.5, 130 mM NaCl) containing 0.4% NP-40. Next, Protein A/G agarose beads (#sc-2003, Santa Cruz Biotechnology) were applied to the lysates and the samples were further treated for 2 hours with rotation. Samples were washed three times with TBS. And proteins were eluted using 2X LDS buffer at room temperature for 30 min. Subsequently, the samples were boiled for 5 minutes and separated using SDS-PAGE.

### 2.8. Immunoprecipitation LC-MS/MS (IP-MS)

After SDS-PAGE, LC-MS/MS was conducted to identify the potential interacting protein of USP2 in cancer cells. Cell samples were divided into multiple fractions. The supernatants of each cell fraction were detected with silver staining. LC-MS/MS experiments were conducted using LTQ Orbitrap Elite (Thermo Fisher) and a Waters NanoAcquity HPLC pump (Milford, MA, USA).

### 2.9. Matrigel Transwell and Invasion Assay

Firstly, 2 × 10^6^ tumor cells from each treatment group were placed in the upper chamber, coated with a matrix gel (#354230, 1 : 8 diluted, Corning, ME) with FBS-free DMEM. Then DMEM containing 10% FBS was placed into the lower chamber as chemoattraction. Mitomycin C was placed in the upper chamber to inhibit cell proliferation. After 24 hours of cell culture, the cells that had invaded the lower membrane surface were fixed by 4% methanol, stained with crystal violet, and counted in 10 random × 100 microscopic fields per sample.

### 2.10. Wound Healing Assay

Each group of cells was placed in six-well culture plates until the confluence reached 90%. After serum starvation for 24 hours, a sterile pipette tip was used to scratch the monolayer. The distance that cells had migrated was photographed by a digital camera under an inverted microscope (Olympus) at the same position at 0 and 48 hours for later calculation. Image-Pro Plus 6.0 software (Media Cybernetics, Bethesda, MD, USA) was used to measure and calculate the distance that the cells had migrated.

### 2.11. Ubiquitination Assay

Cell line samples with a density of 2 × 10^5^ were transferred onto 10 cm cell culture dishes. After 24 h, 25 *μ*M MG132 was added to cultured cells for 6 h. Cell samples were rinsed twice with cold PBS and were lysed using 5 ml of lysis buffer. The cell lysate samples were sonicated twice before adding 50 *μ*L of Ni- NTA agarose beads (QIAGEN, Cat No. 30210). The treated samples were furtherly incubated for another 4 h at 4°C. Next, the beads were purified with 10 mL of denaturing lysis buffer, combined with twice purification in wash buffer B. Then beads were treated with 55 *μ*L of elution buffer for 20 minutes at room temperature and were subsequently centrifuged under 7000 rpm for 3 min. The supernatant was then added with DTT to reach the concentration of 40 mM. Then samples were boiled for further Western blot assay.

### 2.12. Statistical Analysis

All data are presented as the mean ± standard error and analyzed using SPSS software (SPSS Co., Ltd., USA). One-way ANOVA or Student's *t*-test was performed to analyze data. ^∗^ indicates *p* < 0.05, ^∗∗^ indicates *p* < 0.01, ^∗∗∗^ indicates *p* < 0.001, and # indicates no statistical significance. The experiments were repeated at least 3 times.

## 3. Results

### 3.1. USP2 Is Significantly Suppressed and Interacts with ARID2 in Lung Cancer Cells

**Figure 1:**
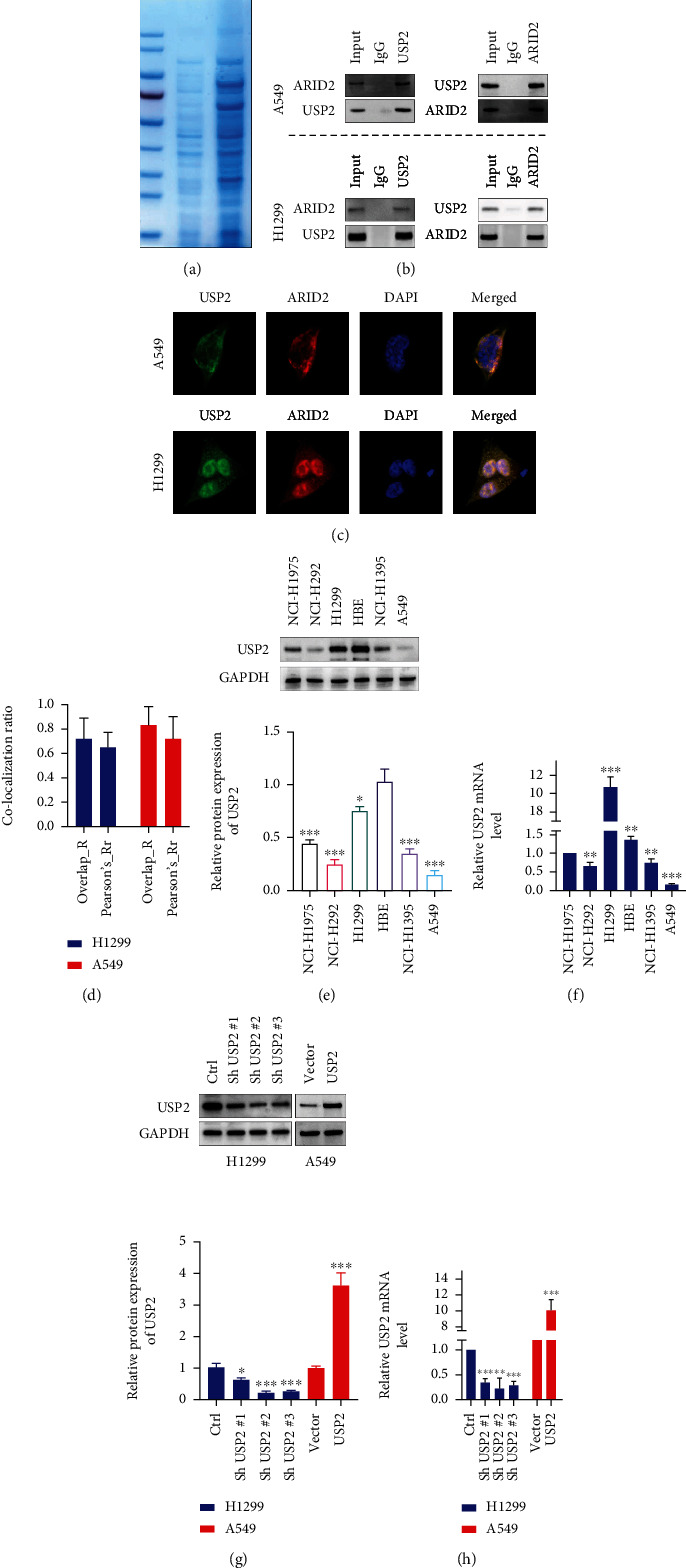
USP2 is significantly suppressed and interacts with ARID2 in lung cancer cells; H1299 cells were transfected with Sh USP2#1, Sh USP2#2, Sh USP2#3, or negative control vector. A549 cells were transfected with USP2 overexpression vector or negative control vector. (a) Coomassie blue staining on H1299 cell line protein samples immunoprecipitated with IgG and anti-USP2 antibody. (b) Validation of USP2-ARID2 interaction by immunoprecipitation method in H1299 and A549 cell lines. (c) Immunofluorescence colocalization method to detect USP2-ARID2 localization in H1299 and A549 cells. (d) Statistical analysis on the colocalization ratio of USP2 and ARID2 using overlap and Pearson's ratio parameters, respectively. (e, f) Expression profile of USP2 protein and mRNA in multiple human lung cancer cell lines (NCI-H1975, NCI-H292, H1299, NCI-H1395, and A549) and normal human bronchial epithelial cell line (HBE) by WB tests (^∗∗^ indicates *p* < 0.01 compared with group HBE; ^∗∗∗^ indicates *p* < 0.001 compared with group HBE). (g, h) Design and validation of USP2-specific shRNAs and USP2-specific overexpression vectors' effects on USP2 mRNA expression in H1299 and A549 cells by PCR methods (^∗∗^ indicates *p* < 0.01 compared with group control; ^∗∗∗^ indicates *p* < 0.001 compared with group control or vector).

In this study, to identify potential interaction protein targets of USP2, IP-MS analysis with H1299 cell line was performed (Figure 1(a)). Results suggested that in comparison of control IgG-precipitated protein levels, several USP2-precipitated proteins, including NPIPA3, CCR8, RTEL1, SKA3, and ARID2, are significantly enriched (Supplementary Figure 1 A). Based on the above findings, we then utilized HEK-293T cell line as a model to explore and confirm the potential interaction of USP2-ARID2. As shown in Supplementary Figure 1 B-C, HEK-293T cell line was transfected with Myc-tagged USP2 vectors, in combination with or without Flag-tagged ARID2. Using anti-Flag or anti-Myc antibody precipitated protein as input, interactive Myc-USP2 protein (Supplementary Figure 1 B) and interactive Flag-ARID2 (Supplementary Figure 1 C) were, respectively, detected; the above findings provided direct evidence of the interaction of USP2 and ARID2 in cell line models. Additionally, immunoprecipitation and immunofluorescence colocalization assays were subsequently applied in A549 and H1299 cancer cell lines. The results indicated that in both A549 and H1299 cells, ARID2 protein can be detected in anti-USP2 antibody precipitated protein samples, and vice versa (Figure 1(b)), suggesting endogenously USP2-ARID2 protein-protein interaction (PPI) in lung cancer cells. We also detected apparent colocalization of USP2 and ARID2 within cancer cells. The quantitative analysis of the USP2-ARID2 colocalization ratio also provided consistent results (Figures 1(c) and 1(d)). Interestingly, USP2 localized in the nucleus in H1299, but not in A549, which needs to be further explored.

To explore the expression and gene regulation features of USP2 in lung cancer, several lung cancer cell lines, including NCI-H1975, NCI-H1292, H1299, NCI-H1395, A549 cells, and normal bronchial epithelial cell line (HBE), were utilized. Through mRNA and protein level detection, we discovered that majority of lung cancer cell lines had suppressed USP2, except for H1299 (Figures 1(e) and 1(f)). USP2-specific shRNAs and overexpression were designed and validated USP2 modulation effects (Figures 1(g) and 1(h)) to further explore the effects of USP2 gene modulation on ARID2 gene expression. The highest expression of USP2 was found in the H1299 cell line, and the lowest expression of USP2 was found in the A549 cell line (Figures 1(e) and 1(f)). Therefore, the H1299 cell line was used to construct knockdown vectors of USP2, and the A549 cell line was used to construct overexpression vectors of USP2.

**Figure 2:**
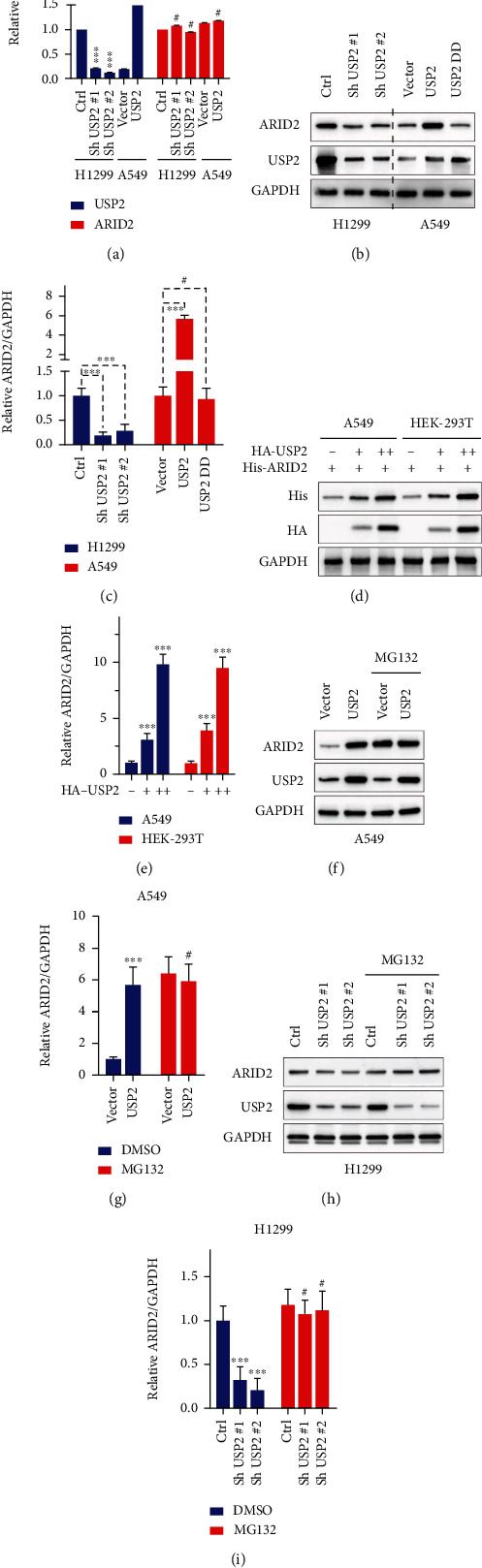
The expression and gene regulation features of USP2 in lung cancer; H1299 cells were transfected with Sh USP2#1, Sh USP2#2, or negative control vector. A549 cells were transfected with USP2 overexpression vector or negative control vector. (a) Modulative effects of USP2-specific shRNAs and overexpression vectors on the mRNA expression level of USP2 and ARID2 detected by qRT-PCR (^∗∗∗^ indicates *p* < 0.001 compared with group control; # indicates no statistical significance). (b, c) Modulative effects of USP2-specific shRNAs and overexpression vectors on the protein expression level of wild-type USP2 and C276R loss-of-function mutated USP2 (^∗∗∗^ indicates *p* < 0.001 compared with negative control vector; # indicates no statistical significance). (d, e) Exogenously transfected USP2 regulated ARID2 protein level in a dose-dependent manner in HEK-29T and A549 cells (“++” sign indicated twice the amount of HA-USP2 vector; ^∗∗∗^ indicates *p* < 0.001 compared with negative control vector). (f, g) ARID2 protein level detection in A549 cells transfected with USP2 overexpression vectors; each group was treated with or without proteasome inhibitor MG132 (^∗∗∗^ indicates *p* < 0.001 compared with negative control vector; # indicates no statistical significance). (h, i) ARID2 protein level detection in H1299 cells transfected with USP-specific shRNAs; each group of cells was treated with or without proteasome inhibitor (^∗∗∗^ indicates *p* < 0.001 compared with negative control vector; # indicates no statistical significance).

RT-PCR detection indicated that after transfection of USP2 shRNAs or overexpression vector, ARID2 mRNA expression level showed no significant change (Figure 2(a)). In comparison, Western blot suggested that after transfection of shRNAs, ARID2 protein level was suppressed. The USP2 overexpression vector transfection enhanced ARID2 protein expression. Notably, transfection of the C276R loss-of-function mutated USP2 vector did not change the level of ARID2 protein expression (Figures 2(b) and 2(c)). The above results suggested that posttranscriptional regulation might be accountable for the regulatory function of USP2 on an ARID2 protein level. Further experiments using HA-tagged USP2 vector transfection suggested that ARID2 mRNA and protein levels were regulated in a dose-dependent manner, relying on the amount of transfected HA-USP2 vectors (Figures 2(d) and 2(e)). To explore the potential role of proteasome-dependent protein degradation in USP2-ARID2 regulation, MG132 was then applied in A549 cells transfected with USP2 overexpression vectors. Western blot results suggested MG132 abrogated the promotive effects of USP2 overexpression on ARID2 protein level (Figures 2(f) and 2(g)). In consistence, in the H1299 cells transfected with USP2 specific-shRNAs, MG132 treatment abrogated the suppressive effects of USP2 silencing on ARID2 protein level (Figures 2(h) and 2(i)).

### 3.2. USP2 Modulated ARID2 Protein Level via Ubiquitination Suppression and Subsequent ARID2 Protein Stabilization

**Figure 3:**
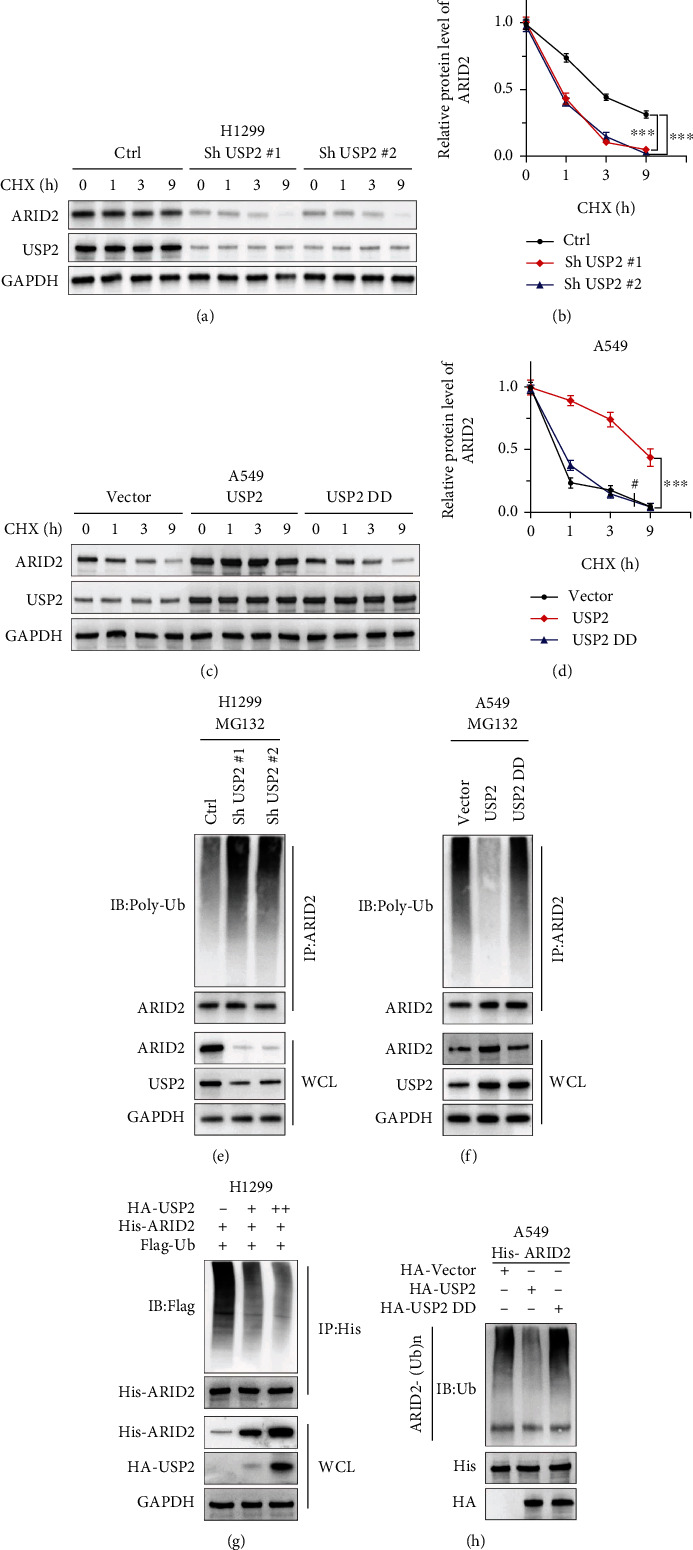
USP2 modulated ARID2 protein level via ubiquitination suppression and subsequent ARID2 protein stabilization; H1299 cells were transfected with Sh USP2#1, Sh USP2#2, or negative control vector. A549 cells were transfected with USP2 overexpression vector or negative control vector. (a, b) CHX treatment of H1299 cell line groups transfected with USP2 shRNAs to evaluate the influences of USP2 expression suppression on ARID2 protein stability and degradation. Relative quantification of ARID2 protein level was analyzed in three groups treated by CHX for 0 h to 9 h (^∗∗∗^ indicates *p* < 0.001 compared with negative control vector). (c, d) CHX treatment of A549 cell line groups transfected with USP2 or loss-of-function mutated USP2 overexpression vectors to evaluate the influences of USP2 overexpression on ARID2 protein stability and degradation. Relative quantification of ARID2 protein level was analyzed in three groups treated by CHX for 0 h to 9 h (^∗∗∗^ indicates *p* < 0.001 compared with negative control vector; # indicates no statistical significance). (e) Ubiquitination analysis using the Co-IP method in H1299 cell line groups transfected with or without USP2 shRNAs. Anti-Poly-Ub antibody was used to detect ubiquitination levels in protein samples immunoprecipitated by the anti-ARID2 antibody. Whole cell lysate samples were used to detect USP2 and ARID2 protein levels. (f) Ubiquitination analysis using the Co-IP method in A549 cell line groups transfected with USP2 or mutated USP2 overexpression vectors. Anti-Poly-Ub antibody was used to detect ubiquitination levels in protein samples immunoprecipitated by the anti-ARID2 antibody. Whole cell lysate samples were used to detect USP2 and ARID2 protein levels. (g) Ubiquitination analysis using the Co-IP method in HEK-293T cell line groups transfected with His-ARID2 and Flag-Ub, in combination with different doses of HA-USP2. Anti-Poly-Ub antibody was used to detect ubiquitination levels in protein samples immunoprecipitated by the anti-His antibody. Whole cell lysate samples were used to detect HA-USP2 and His-ARID2 protein levels. (h) ARID2 ubiquitination analysis using the Co-IP method in HEK-293T cell line groups transfected with His-ARID2, in combination with HA-Vector, HA-USP2, or HA-mutated USP2, respectively. Anti-Ub antibody was used to detect ARID2 ubiquitination level in protein samples immunoprecipitated by anti-ARID2-(Ub) antibody. Whole cell lysate samples were used to detect HA-USP2 and His-ARID2 protein levels.

To understand the role of ubiquitination on USP2 regulative function on ARID2 protein level, protein synthesis inhibitor cycloheximide (CHX) was applied to treat the H1299 cell line. H1299 cells were transfected with USP2 shRNAs or control vectors, respectively. Study results showed that cancer cells transfected with USP2 shRNAs exhibited significantly promoted ARID2 protein degradation compared with a control group (Figures 3(a) and 3(b)). In comparison, in A549 cell line groups transfected with USP2 or loss-of-function mutated USP2 (USP2 DD) overexpression vectors, groups with USP2 overexpression exhibited significantly reduced protein degradation compared with USP2 DD transfection group and control group (Figures 3(c) and 3(d)).

Furthermore, we then aimed to investigate the influence of USP2 expression on the ubiquitination level of ARID2. Co-IP assays were performed on H1299 and A549 cell lines, respectively, transfected with USP2 shRNAs or USP2/USP2 DD overexpression vectors. When whole cell lysate was analyzed, ARID2 protein level was significantly suppressed in USP2 silencing groups, while being significantly increased in the USP2 overexpression group. The result of the anti-ARID2 antibody precipitated protein indicated that USP2 silencing significantly enhanced the ubiquitination level of ARID2 in H1299 cells, while USP2 overexpression significantly reduced the ubiquitination level of ARID2 in A549 cells (Figures 3(e) and 3(f)).

Co-IP assays were performed on H1299 and A549 cell line groups with exogenous transfection of tagged vector His-ARID2, in combination with or without tagged vectors including HA-USP2, HA-USP2 DD, and Flag-Ubiquitin. As shown in Figure 3(g), HA-USP2 vector dose-escalation transfection significantly enhanced His-ARID2 level in whole cell lysate samples, while the Flag-Ubiquitin level was decreased in a dose-dependent manner in anti-His antibody-precipitated input protein samples. Likewise, HA-USP2 transfection significantly reduced ARID2 ubiquitination, while HA-USP2 DD transfection had no significant impact on ARID2 ubiquitination (Figure 3(h)). In summary, the above evidence supported our theory that USP2 enhances cellular ARID2 protein level through its inhibitory function on ARID2 protein degradation via the ubiquitination pathway.

### 3.3. USP2 Significantly Inhibits Lung Cancer Cells through ARID2 Modulation

**Figure 4:**
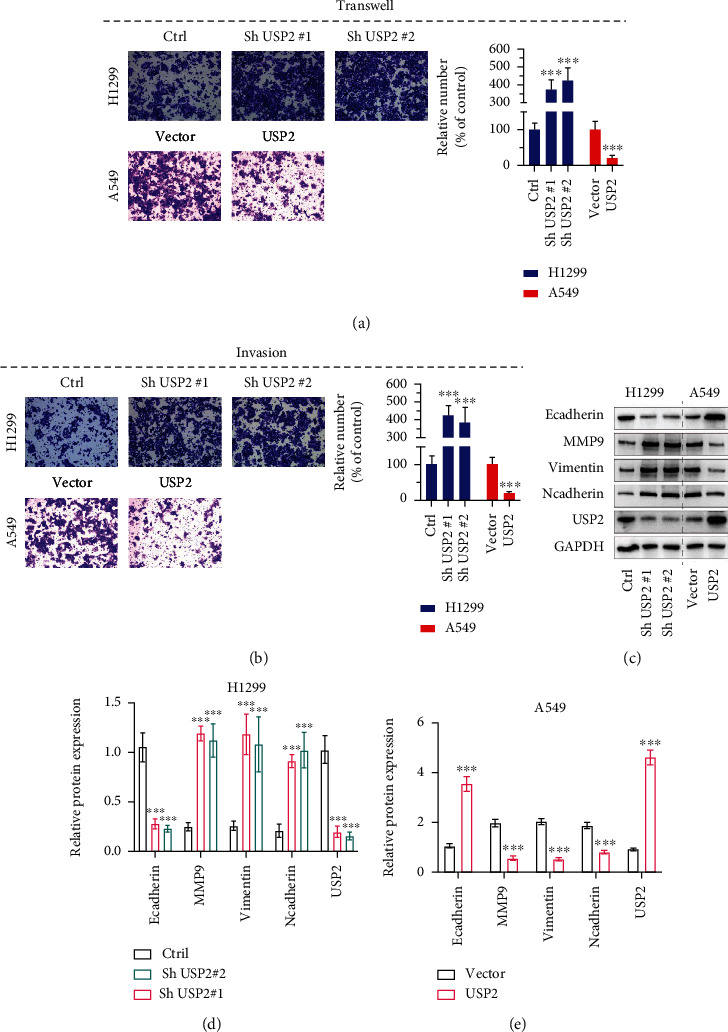
USP2 inhibits lung cancer cell migration and invasion in vitro; H1299 cells were transfected with Sh USP2#1, Sh USP2#2, or negative control vector. A549 cells were transfected with USP2 overexpression vector or negative control vector. (a) Transwell study on H1299/A549 cells transfected with USP2 shRNAs or USP2 overexpression vectors. Relative numbers of migrated cells were calculated in the right chart (^∗∗∗^ indicates *p* < 0.001 compared with negative control vector). (b) Invasion study on H1299/A549 cells transfected with USP2 shRNAs or USP2 overexpression vectors. Relative numbers of invaded cells were calculated in the right chart (^∗∗∗^ indicates *p* < 0.001 compared with negative control vector). (c–e) Tumor cell EMT markers (MMP9, E-Cadherin, N-Cadherin, and Vimentin) and USP2 protein expression value evaluation in H1299 and A549 cell lines transfected with shRNAs or overexpression vectors.

To evaluate the impact of USP2 expression modulation on the malignancy of lung cancer cell lines, Transwell and invasion experiments were subsequently performed on H1299 and A549 cell line models with transfection of USP2 shRNAs or overexpression vectors. As shown in Figures 4(a) and (b), USP2 shRNA transfection uniformly enhanced H1299 and A549 cell migration and invasion, while USP2 overexpression vector transfection significantly suppressed the migrative and invasive capability of cancer cells.

Subsequent wound healing assay suggested that H1299 cells with USP2 shRNAs transfection elevated cancer cell migration and wound healing after 48 h of incubation (Supplementary Figure 2 A-B). In comparison, USP2 overexpression vector-transfected A549 cells demonstrated impaired cellular migration (Supplementary Figure 2 A-B). We utilized Western blot assays to detect the influences of USP2 modulation on biomarkers of cancer cellular epithelial-mesenchymal transition (EMT) (Figures 4(c)–4(e)). Results suggested that USP2 shRNAs transfection notably suppressed E-Cadherin levels, while enhancing the protein level of MMP9, Vimentin, and N-Cadherin levels, and USP2 overexpression vector transfection in A549 cells demonstrated opposite effects. The above findings further suggested that USP2 expression suppression via shRNAs transfection might enhance lung cancer cell migration and invasion by boosting the cellular EMT.

**Figure 5:**
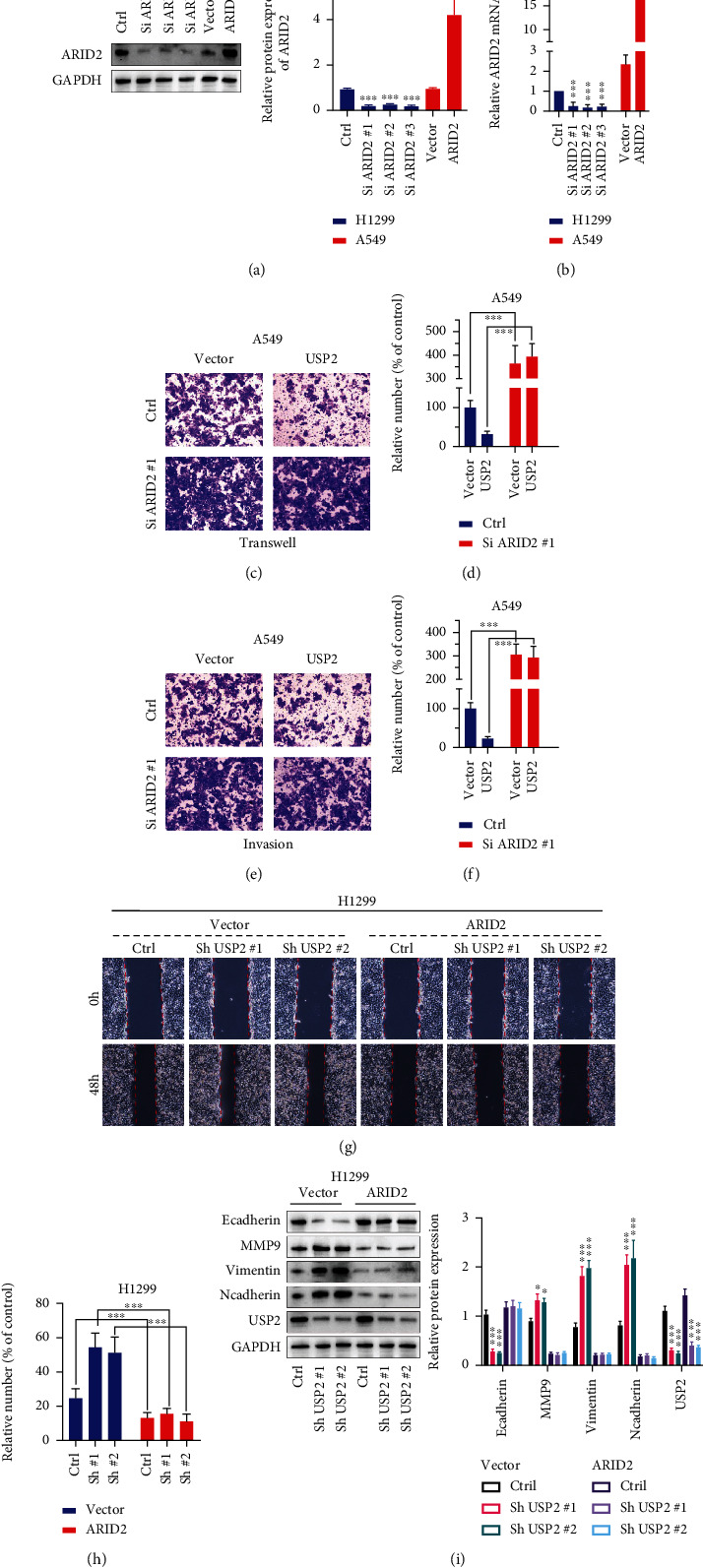
USP2 affects lung cancer cell migration and invasion through ARID2 regulation; H1299 cells were transfected with SiARID2#1, SiARID2#2, SiARID2#3, or negative control vector. A549 cells were transfected with ARID2 overexpression vector or negative control vector. (a, b) Design and validation of ARID2-specific siRNAs and overexpression vectors' effects on the mRNA and protein expression of ARID2 in H1299 and A549 cells (^∗∗∗^ indicates *p* < 0.001 compared with negative control vector). (c, d) Transwell study on A549 cells transfected with ARID2 siRNA in combination with or without USP2 overexpression vectors. Relative numbers of migrated cells were statistically compared (^∗∗∗^ indicates *p* < 0.001 compared with USP2 overexpression vector or negative control vector). (e, f) Tumor cell invasion assay on A549 cells transfected with ARID2 siRNA in combination with or without USP2 overexpression vectors. Relative numbers of invaded cells were statistically compared (^∗∗∗^ indicates *p* < 0.001 compared with USP2 overexpression vector or negative control vector). (g, h) Wound healing assay on H1299 cell line groups transfected with USP2 shRNAs in combination with or without ARID2 overexpression vectors. Relative numbers of migrative cells were statistically compared (^∗∗∗^ indicates *p* < 0.001). (i) Tumor cell EMT markers (MMP9, E-Cadherin, N-Cadherin, and Vimentin) and USP2 protein expression value evaluation in H1299 cell lines groups transfected with USP2 shRNAs in combination with or without ARID2 overexpression vectors.

To confirm the above-mentioned theories, ARID2-specific shRNAs and overexpression vectors were further designed and validated (Figures 5(a) and 5(b)). Transwell and invasion assay was conducted on A549 cell line groups with USP2 overexpression vector transfection, in combination with or without ARID2-siRNAs or overexpression vector transfection. As shown in Figures 5(c) and 5(d), when ARID2-siRNAs combined with USP2 overexpression vectors were cotransfected, the suppressive effects of USP2 overexpression on A549 cell migrated capabilities were abrogated. Consistent results were also observed in invasion assay evaluation in A549 cells, respectively, transfected with USP2 overexpression vectors in combination with or without ARID2 siRNAs (Figures 5(e) and 5(f)). In summary, silencing ARID2 alone in A549, irrespective of USP2 expression status, enhanced cell migration and invasion. Subsequent wound healing assay on the H1299 cells transfected with USP2 shRNAs in combination with or without ARID2 overexpression vectors also suggested that the enhancement of cancer cell migration caused by USP2 shRNAs was also diminished with cotransfection of ARDI2 overexpression (Figures 5(g) and 5(h)). Therefore, in H1299 cells, overexpression of ARID2 decreased cell migration, irrespective of USP2 status. When EMT biomarkers were detected in H1299 cells with cotransfection of USP2 shRNAs and ARID2 overexpression vectors, we found that in comparison with cancer cells with USP2 shRNAs transfection, the cotransfection demonstrated notably diminished tendency of mesenchymal phenotype transition (Figure 5(i)). To summarize, we confirmed that USP2 suppressed cancer cell migration and invasion capabilities through ARID2.

### 3.4. ARID2 Expression Is Positively Correlated with USP2 Expression in Clinical Samples Tissues

**Figure 6:**
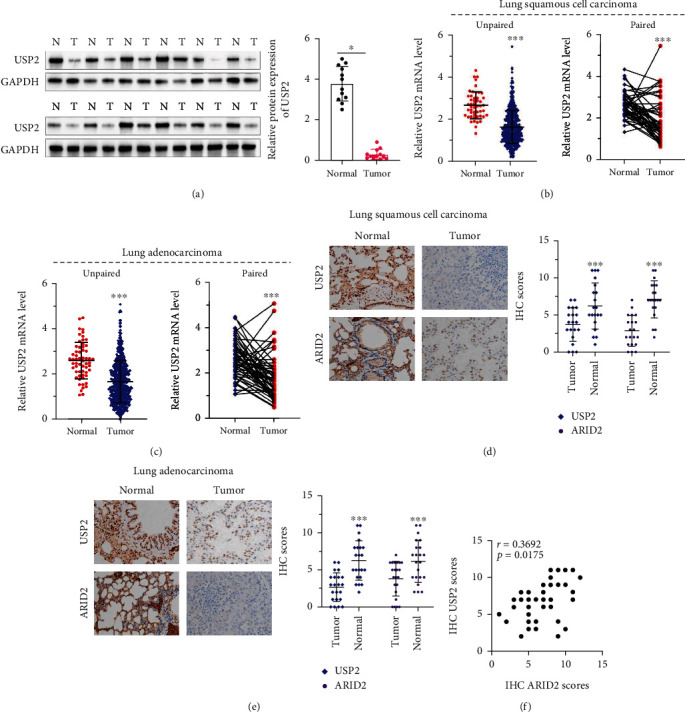
ARID2 expression is positively correlated with USP2 expression in lung cancer tissues. (a) WB assay on USP2 expression in paired lung cancer tumor tissues and normal tissues adjacent to tumor. (b, c) TCGA dataset analysis on the USP2 mRNA expression in paired lung squamous cell cancer/lung adenocarcinoma tissues and normal lung tissues (^∗∗∗^ indicates *p* < 0.001 compared with group normal). (d–f) Immunohistochemistry assay to evaluate USP2 and ARID2 expression levels in paired samples of lung cancer tumor and normal tissues adjacent to tumor (d). Semiquantitative values of USP2 and ARID2 expression levels were calculated and statistically compared between cancer and normal tissues (e), and the correlation of USP2 and ARID2 expression levels were also examined (f) (^∗∗∗^ indicates *p* < 0.001 compared with group tumor).

To validate our findings of the USP2-ARID2 regulatory axis in lung cancer cell line models, USP2 protein and mRNA expression were further detected in paired and unpaired clinical lung cancer and normal tissue (NT). As shown in Figure 6(a), in comparison with normal tissues adjacent to tumor, most tumor tissues exhibited significantly suppressed USP2 protein levels. Moreover, TCGA gene expression profiling datasets of lung squamous cell carcinoma (LSCC) and lung adenocarcinoma tumor samples were utilized to validate the pattern of USP2/ARID2 mRNA expression. Results indicated that both paired and unpaired LSCC and adenocarcinoma tissues demonstrated a significantly lower level of USP2 mRNA expression, compared with NT (Figures 6(b) and 6(c)).

Additionally, immunohistochemistry assay and quantitative statistical analysis results of USP2 and ARID2 expression patterns also suggested that both LSCC and adenocarcinoma cancer tissues exhibited consistently decreased USP2/ARID2 protein expression, compared with NT (Figures 6(d) and 6(e)), and with statistically significant correlation (Figure 6(f)).

## 4. Discussion

In this study, we discovered that the expression of USP2 was significantly suppressed in lung cancer cell models and clinical tumor tissues, in comparison to NT. As previously reported, USP2 has been detected in multiple kinds of human organs and it has been suggested to participate in the pathogenesis of various malignancies. Intriguingly, in contrast with our findings, prior research based on breast cancer tissues and cell line models indicated overexpression of USP2 in metastatic tumor tissues. USP2 was associated with enhanced tumor invasiveness via modulation of MMP2 expression [23]. Another study also suggested that in ErbB2-positive breast cancer, combinative treatment of USP2 inhibitor and Erb2 inhibitor HSP90 demonstrated synergistic therapeutic efficacy [24]. Therefore, the exact roles of USP2 in tumor pathogenesis could be complex and tumor-type-dependent. Comprehensive research of USP2 in lung cancer will be warranted to the full picture of its roles in lung cancer pathogenesis and progression.

In our study, we demonstrated evidence of direct PPI of USP2 and ARID2. USP2 exhibited inhibitory effects on lung cancer cell malignancy via ARID2 de-ubiquitination and protein degradation inhibition. As is well established, ARID2 is a member of the transcriptional coactivator SWI/SNF complexes family. Several studies have indicated the tumor-suppressive function of ARID2 in tumor pathogenesis [25–27]. It has been reported that oncogenic ARID2 loss-of-function mutation is associated with BRG1/BRM-associated factors (BAF) and polybromo-associated BAF (PBAF) complexes (also known as mammalian SWI/SNF complexes) dysfunction. ARID2 and PBAF complex have been indicated as important regulators in cellular DNA damage response (DDR) [28]. Another study demonstrated that ARID2 is involved in cancer cellular immunotherapy resistance [29], which suggested that ARID2 might also regulate T cell cytotoxicity in the tumor microenvironment. The results of our study further expand the regulatory function of ARID2 in lung cancer cell invasiveness and EMT process and suggest the potential of the USP2-ARID2 axis as a novel target in future therapy development for lung cancer. A previous study indicated that ARID2 deficiency could damage DNA repair and enhance the sensitivity of the cells to DNA-damaging agents, and ARID2 played a tumor suppressor role in lung cancer progression [22]. In this study, we demonstrated that knockdown of ARID2 greatly promoted the migration and invasion of lung cancer cells, which is in line with previous research.

Ubiquitination plays a key role in several types of cancers by affecting posttranslational modifications [30]. The regulation of some protein ubiquitination by USP2 in different kinds of tumors has been reported [31, 32]. In addition, a poor prognosis and chemoresistance of multiple myeloma patients were believed to be closely related to the ubiquitination of ARID2 [33]. However, if USP2 could affect tumor progression through targeting ARID2 has not been reported. In this study, we first demonstrated that USP2 inhibited the malignancy of lung cancer by reducing ARID2 protein degradation via ubiquitination.

## 5. Conclusion

In summary, in this study, through lung cancer cell models and clinical sample analysis, we demonstrated for the first time that decreased USP2 expression pattern was a feature in lung cancer cells. USP2 significantly inhibited lung cancer by interacting with ARID2 and reducing ARID2 protein degradation via ubiquitination. Our study suggested the complexity of the USP2-related ubiquitination regulatory network in carcinogenesis, which will enlighten future research on USP2's function in lung cancer.

## Data Availability

The data support this study could be requested from the corresponding author.

## Supplementary Materials

Supplementary Materials Supplementary figure 1: the potential interaction of USP2 and ARID2 was validated. A: Co-IP-MS analysis results of the potential USP2-interacting protein targets. B, C: Co-IP validation experiments on the interaction of USP2 and ARID2. HEK-293 cell line groups were transfected with Flag-ARID2 and/or Myc-USP2 vectors. Anti-Myc and Anti-Flag antibodies were used to detect USP2 and ARID2 protein levels in the whole cell lysate (WCL) or the Flag/Myc specific antibody-immunoprecipitated protein sample. Supplementary figure 2: wound healing assay was performed to investigate the influence of USP2 on cell migration. A, B: wound healing assay on H1299 and A549 cell line groups transfected with USP2 shRNAs or USP2 overexpression vectors. Relative migration ratios of tumor cells in each treatment group were then statistically compared (^∗∗∗^ indicates *p* < 0.001 compared with group control or vector).
