# Supplementary material for: USP2 Inhibits Lung Cancer Pathogenesis by Reducing ARID2 Protein Degradation via Ubiquitination
Source: Biomed Res Int. 2022 Dec 15;2022:1525216. doi: 10.1155/2022/1525216 (PMC9779997; doi:10.1155/2022/1525216)
Supplement: Supplementary Materials — Supplementary figure 1: the potential interaction of USP2 and ARID2 was validated. A: Co-IP-MS analysis results of the potential USP2-interacting protein targets. B, C: Co-IP validation experiments on the interaction of USP2 and ARID2. HEK-293 cell line groups were transfected with Flag-ARID2 and/or Myc-USP2 vectors. Anti-Myc and Anti-Flag antibodies were used to detect USP2 and ARID2 protein levels in the whole cell lysate (WCL) or the Flag/Myc specific antibody-immunoprecipitated protein sample. Supplementary figure 2: wound healing assay was performed to investigate the influence of USP2 on cell migration. A, B: wound healing assay on H1299 and A549 cell line groups transfected with USP2 shRNAs or USP2 overexpression vectors. Relative migration ratios of tumor cells in each treatment group were then statistically compared (∗∗∗ indicates p < 0.001 compared with group control or vector). [file 1525216.f1.docx]

**Supplementary figure 1 The potential interaction of USP2 and ARID2 was validated.** A: Co-IP-MS analysis results of the potential USP2-interacting protein targets. B-C: Co-IP validation experiments on the interaction of USP2 and ARID2. HEK-293 cell line groups were transfected with Flag-ARID2 and/or Myc-USP2 vectors. Anti-Myc and Anti-Flag antibodies were used to detect USP2 and ARID2 protein levels in the whole cell lysate (WCL) or the Flag/Myc specific antibody-immunoprecipitated protein sample.

**Supplementary figure 2 Wound healing assay was performed to investigate the influence of USP2 on cell migration.** A-B: Wound healing assay on H1299 and A549 cell line groups transfected with USP2 shRNAs or USP2 overexpression vectors. Relative migration ratios of tumor cells in each treatment group were then statistically compared (*** indicates p<0.001 compared with group control or vector).
